# Supplementary figures and images for: Temporal analysis of melanogenesis identifies fatty acid metabolism as key skin pigment regulator
Source: PLoS Biol. 2022 May 18;20(5):e3001634. doi: 10.1371/journal.pbio.3001634 (PMC9116682; doi:10.1371/journal.pbio.3001634)

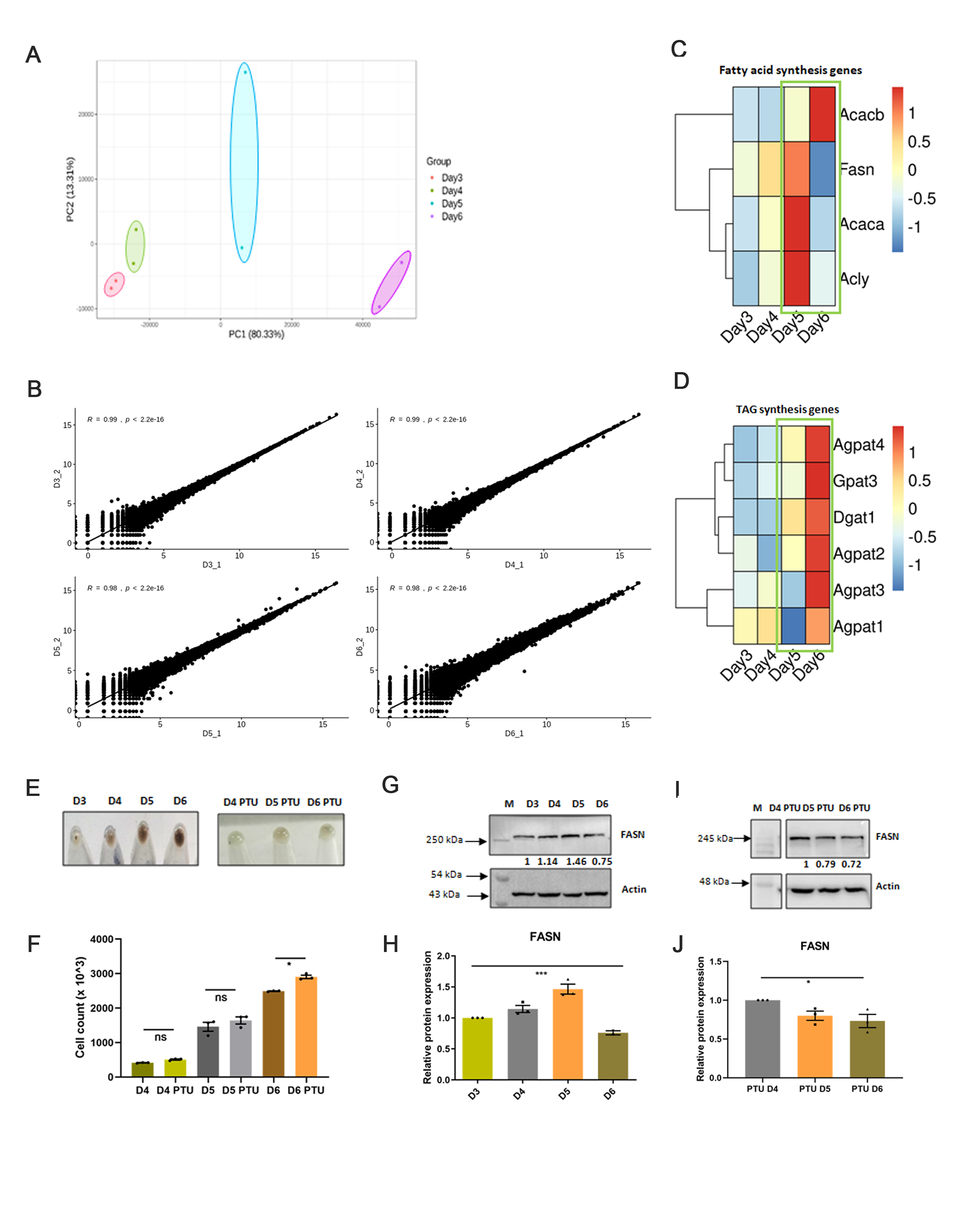

Supplement: S1 Fig — (A) PCA plot from data obtained from unbiased transcriptomic analysis from 2 independent biological replicates. PC1 is 80.33% and PC2 is 13.81%. (B) Correlation plot showing correlation value R obtained between 2 biological replicates in RNA sequencing data. R ranges from 0.99–0.1, with high p-value 2.2e-16. (C) Heatmap representing the expression of fatty acid synthesis genes from days 3 to 6 in RNA sequencing data. Scale from blue to red represents z-score for normalized expression values from ‒1 to +1. (D) Heatmap representing the expression of TAG synthesis genes from days 3 to 6 in RNA sequencing data. Scale from blue to red represents z-score for normalized expression values from ‒1 to +1. (E) Representative image showing pigmented cell pellet in the low-density B16 pigmentation model for days 3 to 6 vs. PTU-treated depigmented cells. (F) Bar graph depicting cell count from days 4 to 6 in control and PTU-treated low-density cells in 3 independent biological sets. One-way ANOVA is applied, F(5,12) = 203.3. Turkey’s test is performed for pairwise comparison. *p-Value < 0.0142. (G) Representative western blot for FASN expression on D3–D6 during melanogenesis with respect to Actin. Numerical values show average fold change for 3 biological replicates. (H) Bar graph depicting quantitative analysis of FASN during melanogenesis for 3 independent biological replicates. Mean ± SEM is plotted for 3 independent biological replicates. One-way ANOVA is applied F(3,7) = 25.74, p-value = 0.0004. (I) Representative western blot for FASN expression on D4–D6 PTU-treated cell-seeded at low density with respect to Actin. Numerical values show average fold change for 3 biological replicates. (J) Bar graph depicting quantitative analysis of FASN during melanogenesis for 3 independent biological replicates. One-way ANOVA is applied F(2,6) = 5.288, p-value = 0.0474. Quantitative data are provided in S2 Data for Panels A, B, F, H, and J. PCA, principal component analysis; PTU, 1-pheny [file pbio.3001634.s001.tif]

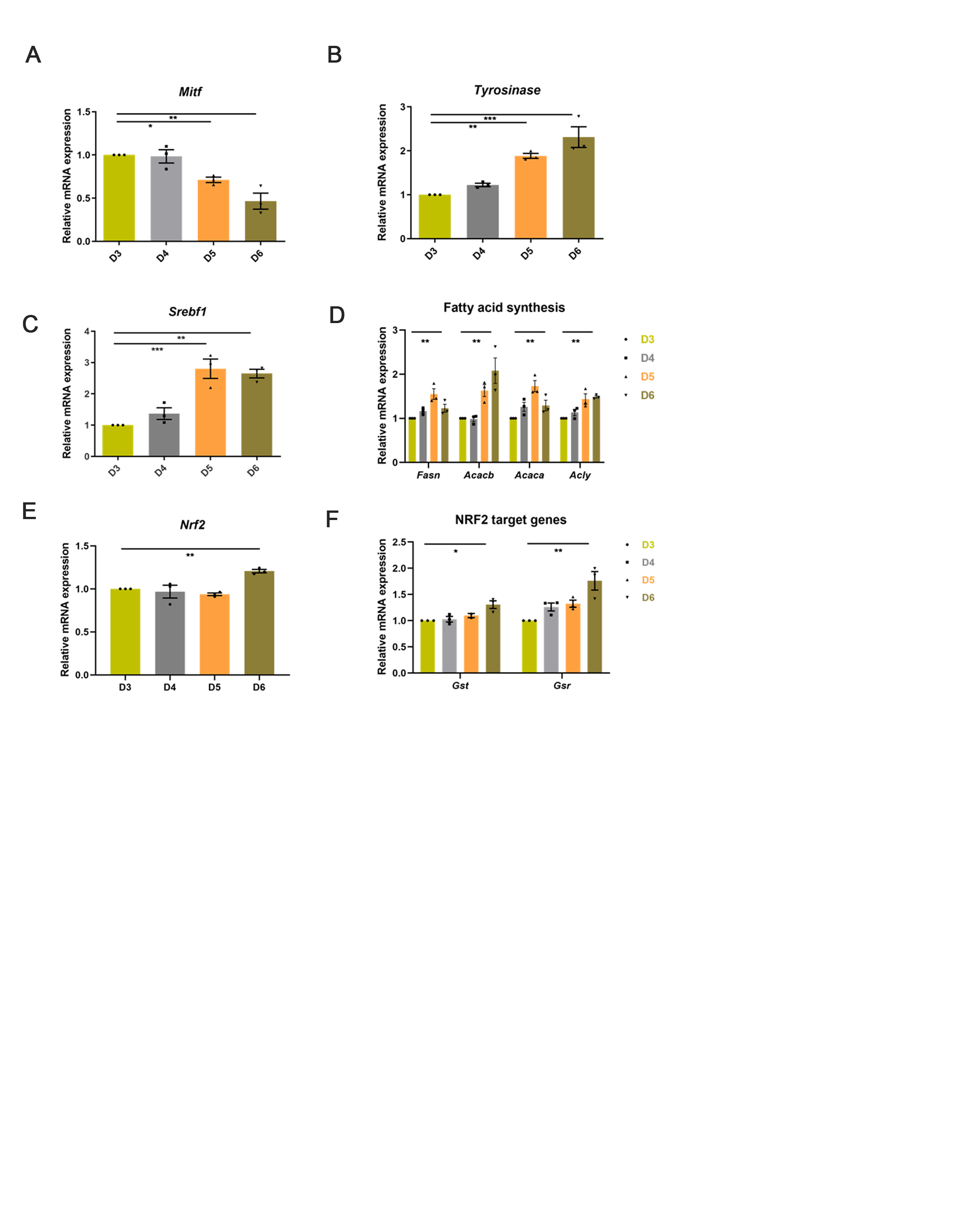

Supplement: S2 Fig — (A) Bar graph depicting qRT-PCR of Mitf with respect to Hgprt. Mean ± SEM is plotted for 3 independent biological replicates. One-way ANOVA is applied. For Mitf, F (3,8) = 16.59, p-value = 0.0009. Turkey’s test is performed for pairwise comparison. (B) Bar graph depicting qRT-PCR of Tyr with respect to Hgprt. Mean ± SEM is plotted for 3 independent biological replicates. One-way ANOVA is applied. For Tyr, F (3,8) = 24.08, p-value = 0.0002. Turkey’s test is performed for pairwise comparison. (C) Bar graph depicting qRT-PCR of Srebf1 with respect to Hgprt. One-way ANOVA is applied. ForSrebf1, F (3,8) = 21.72, p-value = 0.0003. Turkey’s test is performed for pairwise comparison. (D) Bar graph depicting qRT-PCR-based analysis fatty acid synthesis genes, Fasn, Acaca, Acacb, and Acly, with respect to Hgprt. Mean ± SEM is plotted for 3 independent biological replicates. One-way ANOVA is applied separately for each gene. For Fasn F (3,8) = 8.435, p-value = 0.0074, for Acaca F (3,8) = 9.811, p-value = 0.0063, for Acacb F (3,8) = 10.72, p-value = 0.0035, for Acly F (3,8) = 11.06, p-value = 0.0032. (E) Bar graph depicting qRT-PCR of Nrf2 with respect to Gapdh. Mean ± SEM is plotted for 3 independent biological replicates. One-way ANOVA is applied. For Nrf2, F (3,8) = 9.622, p-value = 0.005. Turkey’s test is performed for pairwise comparison. (F) Bar graph depicting qRT-PCR-based analysis Nrf2 TGs, Gsr and Gst, with respect to Gapdh. Mean ± SEM is plotted for 3 independent biological replicates. One-way ANOVA is applied separately for each gene. For Gst, F (3,7) = 7.68, p-value = 0.0128, for Gsr, F (3,8) = 9.666, p-value = 0.0049. Quantitative data are provided in S2 Data for Panels A–F. qRT-PCR, quantitative real-time polymerase chain reaction; TG, target gene. (TIF) [file pbio.3001634.s002.tif]

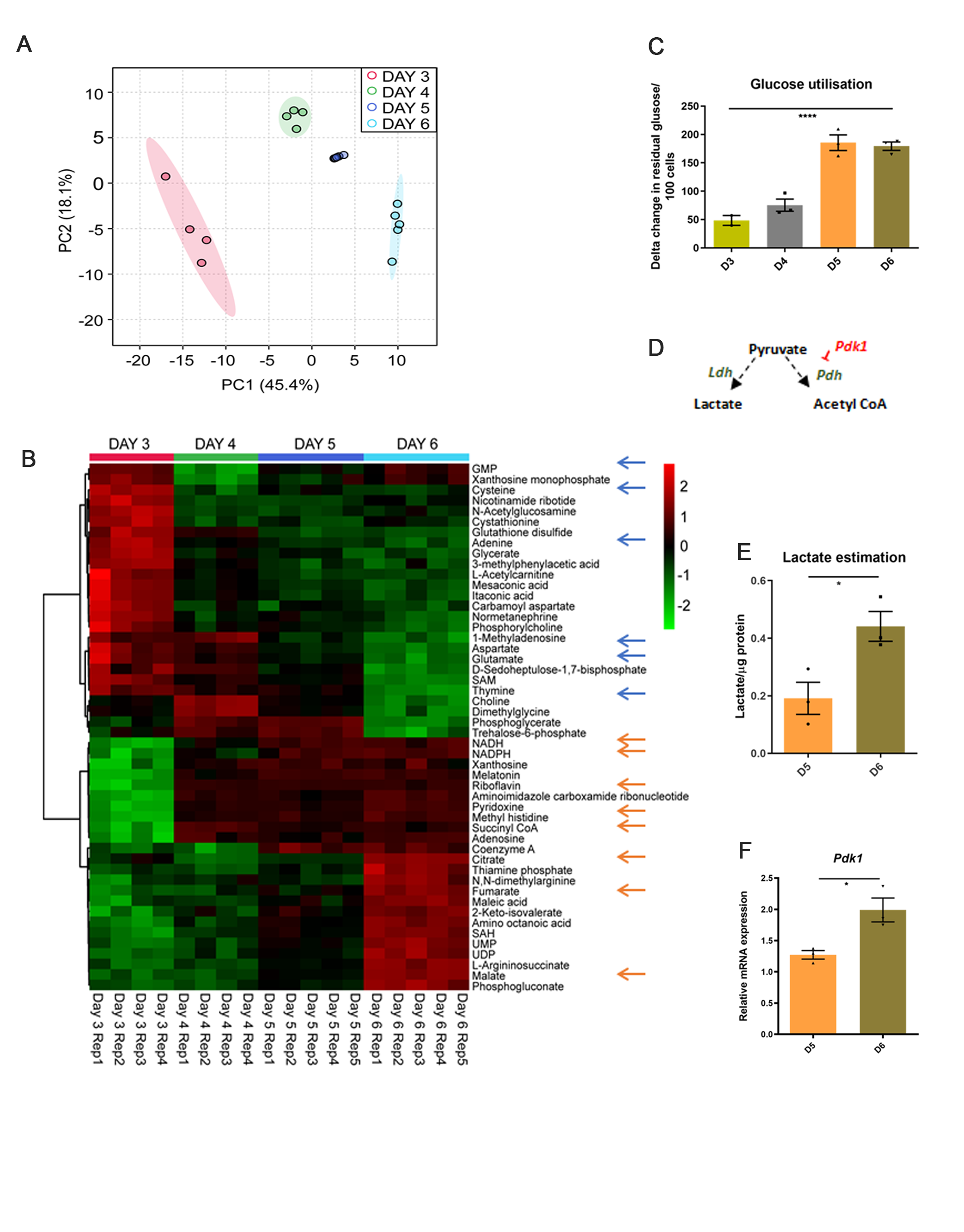

Supplement: S3 Fig — (A) PCA plot depicting segregation of different days based on metabolite signatures. Groups 1 to 4 correspond to days 3 to 6. Four biological replicates are taken for each time point. PC1 is 48.9% and PC2 is 18.5%. (B) Heatmap depicting top 50 regulated metabolites across different days for each replicate. Blue colour arrows are used to show amino acids and nucleotides, higher on D3 and D4, while orange arrows are shown to mark cofactors and TCA metabolites, higher on D5 and D6. (C) Bar graphs depicting glucose utilization upon pigmentation induction in B16 cells cultured at low-density model. Glucose utilization is calculated by subtracting glucose in media on consecutive days (i.e., glucose concentration on (n-1) day-glucose concentration on nth day)/cell number on nth day*100. Data are represented for 3 independent replicates. Mean ± SEM is plotted for 3 biological replicates. One-way ANOVA is applied F(3,7) = 39.50, ****p-value < 0.0001. (D) Schematic showing biochemical reactions and enzymes involved in conversion of pyruvate to lactate and acetyl CoA formation. (E) Bar graph depicting quantitation of amount of lactate in cellular lysate on D5 and D6 for 3 independent replicates. Mean ± SEM is plotted for 3 biological replicates. Two-tailed Student t test is applied, t = 3.284, df = 4, *p-value = 0.0304. (F) Bar graph depicting qRT-PCR-based mRNA expression of Pdk1, which regulates PDH activity on D5 and D6 for 3 independent replicates. Mean ± SEM is plotted for 3 biological replicates. Two-tailed Student t test is applied, t = 3.541, df = 4, *p-value = 0.0240. Quantitative data are provided in S2 Data for Panels C, E, and F. PCA, principal component analysis; qRT-PCR, quantitative real-time polymerase chain reaction; TCA, tricarboxylic acid cycle. (TIF) [file pbio.3001634.s003.tif]

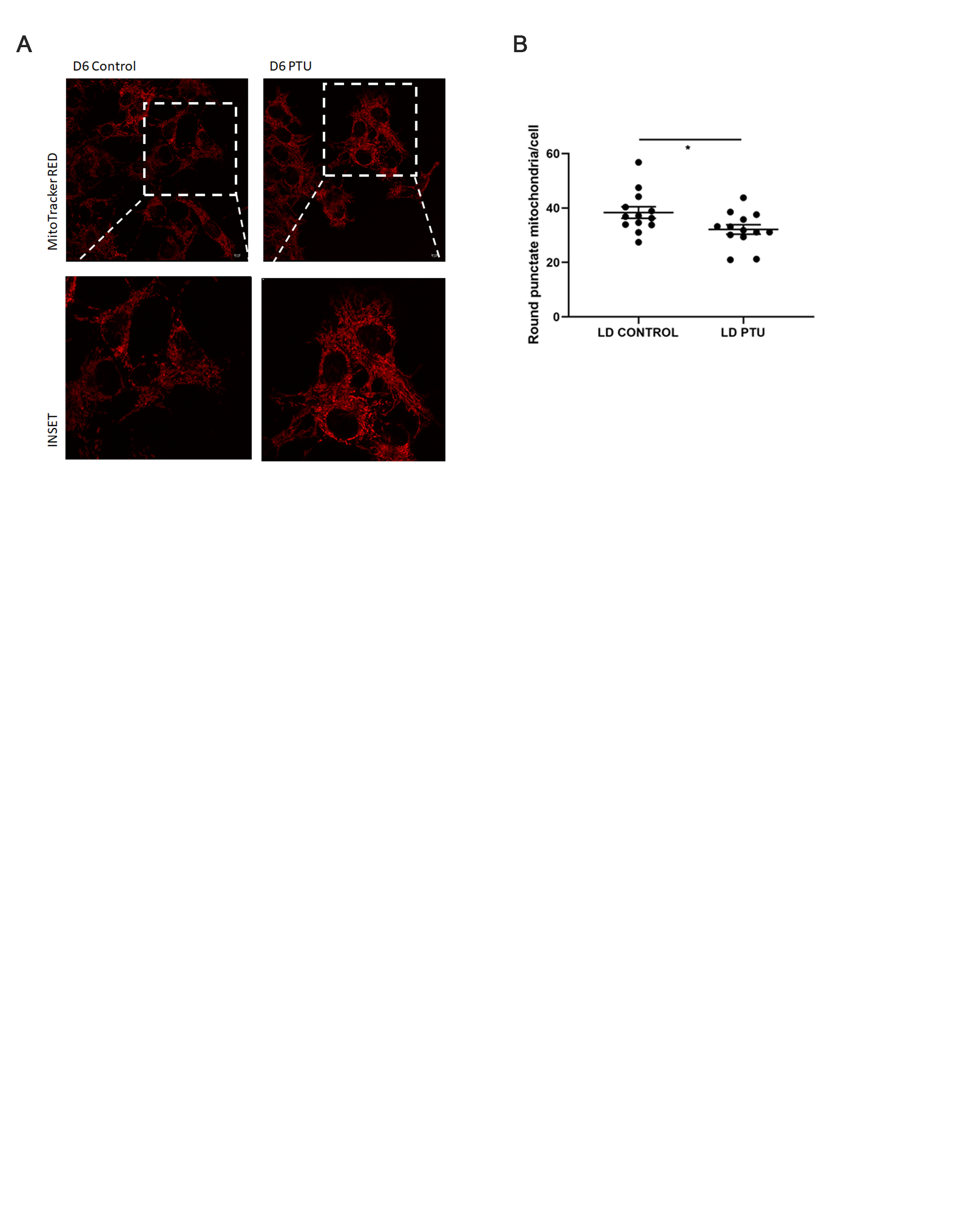

Supplement: S4 Fig — (A) Representative confocal microscopy images showing mitochondrial morphology in pigmented day 6 cells vs. PTU-treated depigmented cells on day 6 using MitoTracker RED dye. Images were taken at 63×. Scale is 5 μm. (B) Bar graph depicting the quantitation of fragmented mitochondria in pigmented vs. PTU-treated depigmented day 6 cells using ImageJ macro tool MiNA. Approximately 100 cells were taken in each replicate. Mean ± SEM is plotted in 3 independent biological replicates. Mean ± SEM is plotted for 3 biological replicates. Two-tailed Student t test is applied, t = 2.262, df = 24, *p-value = 0.0330. Quantitative data are provided in S2 Data for Panel B. MiNA, Mitochondrial Network Analysis; PTU, 1-phenyl-2-thiourea. (TIF) [file pbio.3001634.s004.tif]

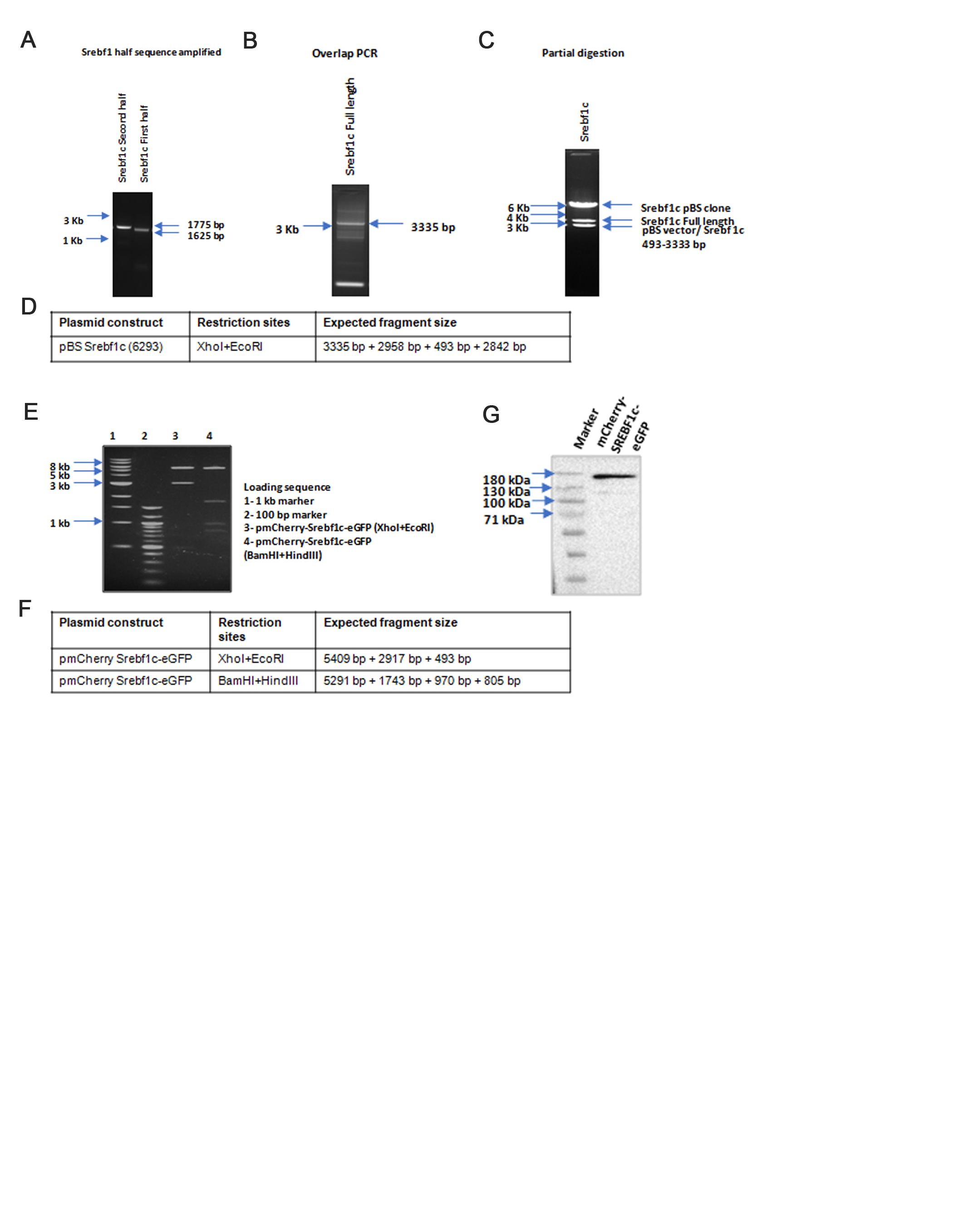

Supplement: S5 Fig — (A) Representative agarose gel showing amplification of first and second half of Srebf1 gene. (B) Representative agarose gel showing amplification of full-length Srebf1 using overlap PCR. (C) Representative agarose gel showing partial digestion of Srebf1-pBS clone, Srebf1 to obtain full-length fragment for subcloning in mCherry-C1 eGFP vector. (D) Expected size of Srebf1 pBS clone is tabulated upon digestion with XhoI and EcoRI. (E) Representative agarose gel showing clone confirmation of mCherry-Srebf1-eGFP vector. (F) Expected size of mCherry-Srebf1-eGFP vector is tabulated upon restriction digestion. (G) Representative western blot for expression analysis of mCherry-Srebf1-eGFP construct upon transfection in B16 cells. (TIF) [file pbio.3001634.s005.tif]

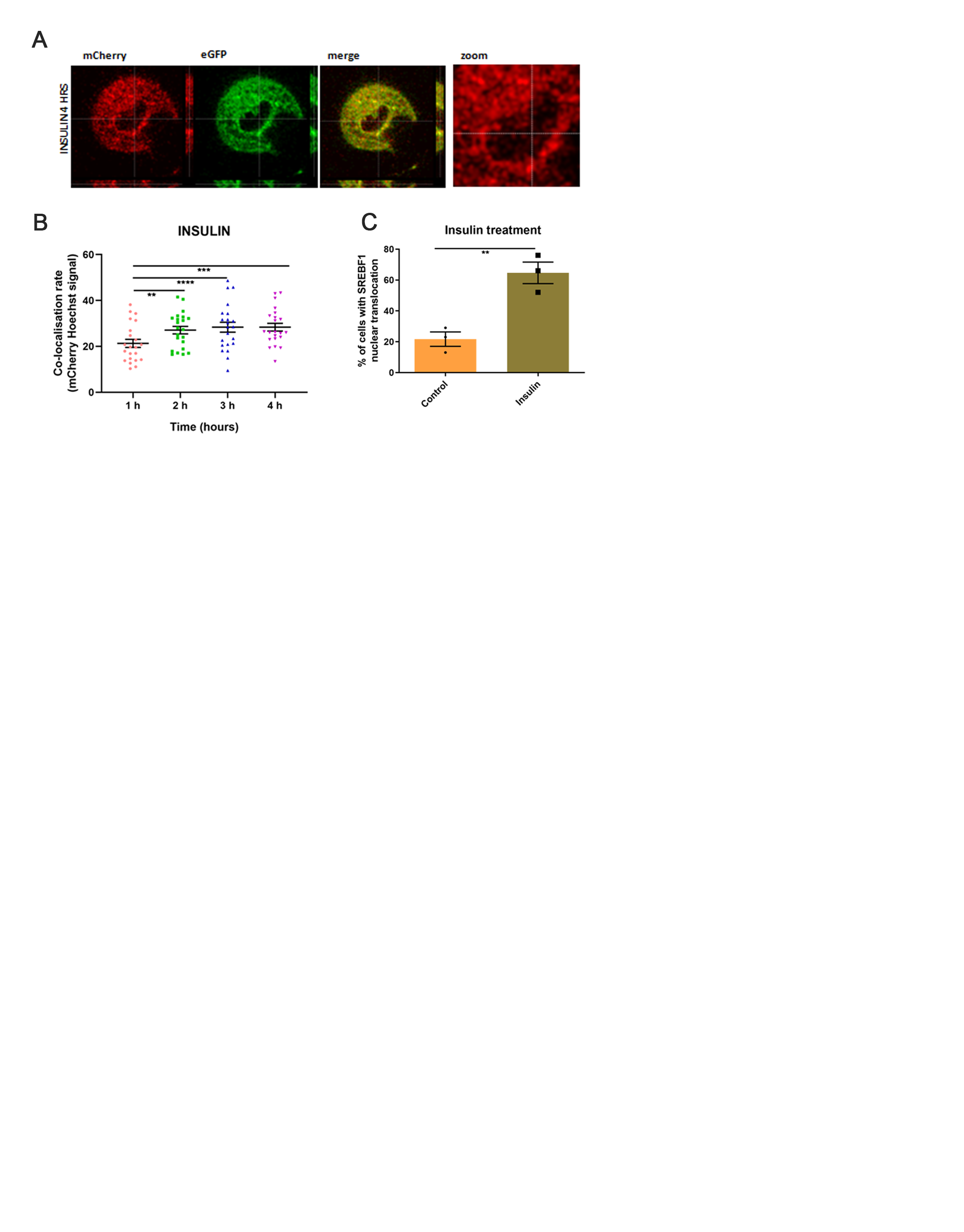

Supplement: S6 Fig — (A) Representative fluorescence images showing eGFP, mCherry, and merge signal. Magnified nuclear images were shown to focus on SREBF1 nuclear translocation after 4 hours of insulin treatment. Around 20 cells were analyzed in each of the 3 biological replicates. (B) Dot plot depicting colocalization rate between mCherry and Hoechst signal analyzed for each cell at 1 to 4 hours after insulin treatment. Mean ± SEM is plotted for 3 biological replicates. One-way ANOVA is applied, F(2.516,52.83) = 8.234, p-value = 0.0003. Dunnett’s multiple comparison test is performed. ****p-Value < 0.0001, ***p-value = 0.00081, **p-value = 0.0011. (C) Bar graph depicting quantitation of number of cells showing positive phenotype after Insulin treatment, determined by increased colocalization rate of mCherry and Hoechst signal from 1 to 4 hours. Mean ± SEM is plotted for combined analysis. Two-tailed Student t test is performed, t = 5.131, df = 4, **p-value = 0.0068. Quantitative data are provided in S2 Data for Panels B and C. (TIF) [file pbio.3001634.s006.tif]

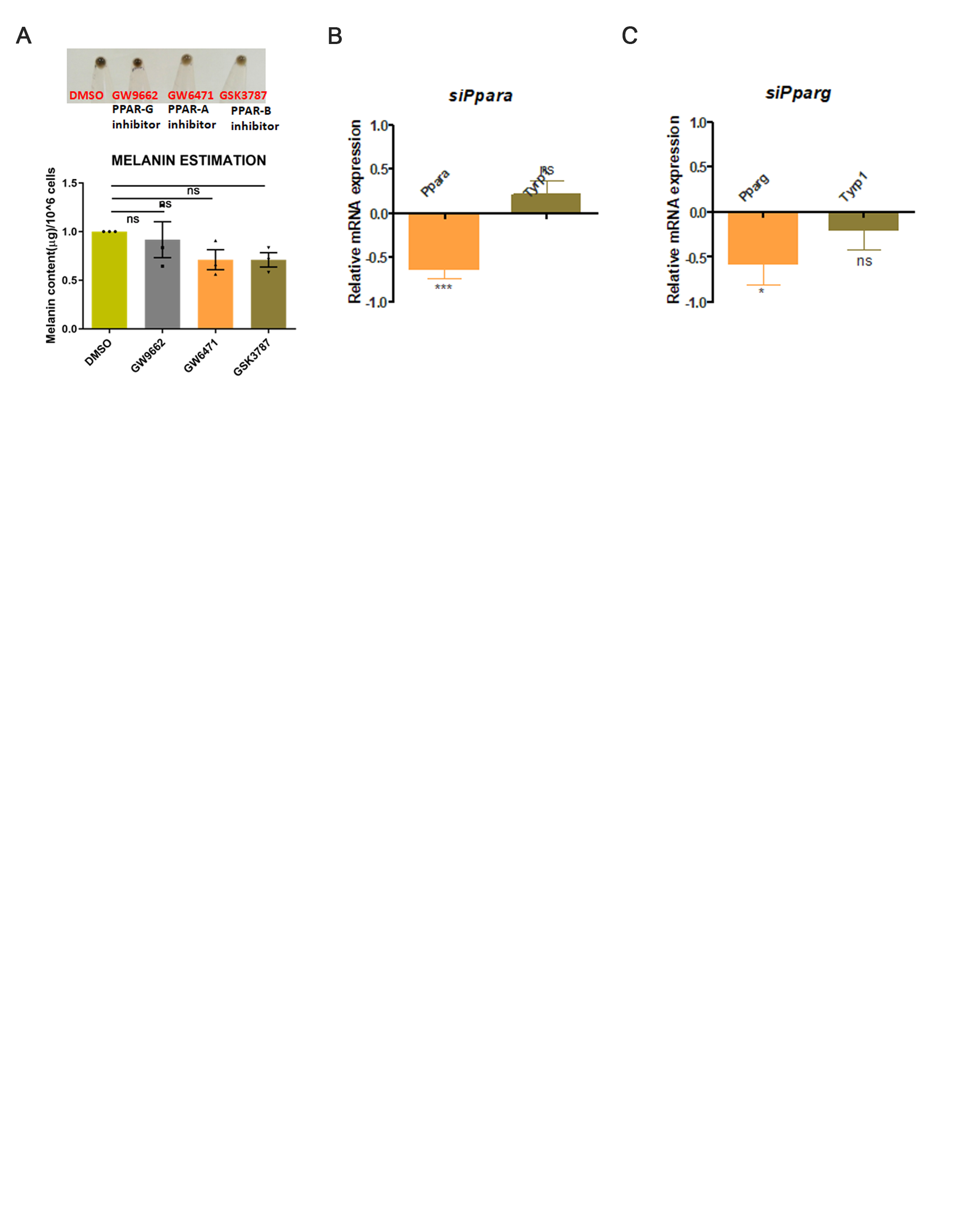

Supplement: S7 Fig — (A) Representative B16 cell pellet images showing melanin accumulation phenotype on day 6 upon inhibiting PPAR-γ with GW9662, PPAR-α with GW6471, and PPAR-β with GSK3787 (N = 3). Bar graph depicting melanin estimation after different inhibitor treatments. Mean ± SEM is plotted for 3 biological replicates. One-way ANOVA is applied, F(3,8) = 1.728. p-Value = 0.2383. Tukey’s test is performed for pairwise analysis. ns is nonsignificant. (B) Bar graph representing qRT-PCR-based quantitation of Ppara and Tyrp1 genes on D5 upon silencing of Ppara using smart pool siRNA. Mean ± SEM is plotted for 3 biological replicates One-way ANOVA is applied, F(3,6) = 49.29. For NT vs. Ppara, ***p-value = 0.0002. For NT vs. Tyrp1, p-value is nonsignificant. (C) Bar graph representing qRT-PCR-based quantitation of Pparg and Tyrp1 genes on D5 upon silencing of Pparg using smart pool siRNA. Mean ± SEM is plotted for 3 biological replicates. One-way ANOVA is applied, F(3,6) = 1.188. For NT vs. Ppara, *p-value = 0.0255. For NT vs. Tyrp1, p-value is nonsignificant. Quantitative data are provided in S2 Data for Panels A–C. qRT-PCR, quantitative real-time polymerase chain reaction; siRNA, small interfering RNA. (TIF) [file pbio.3001634.s007.tif]

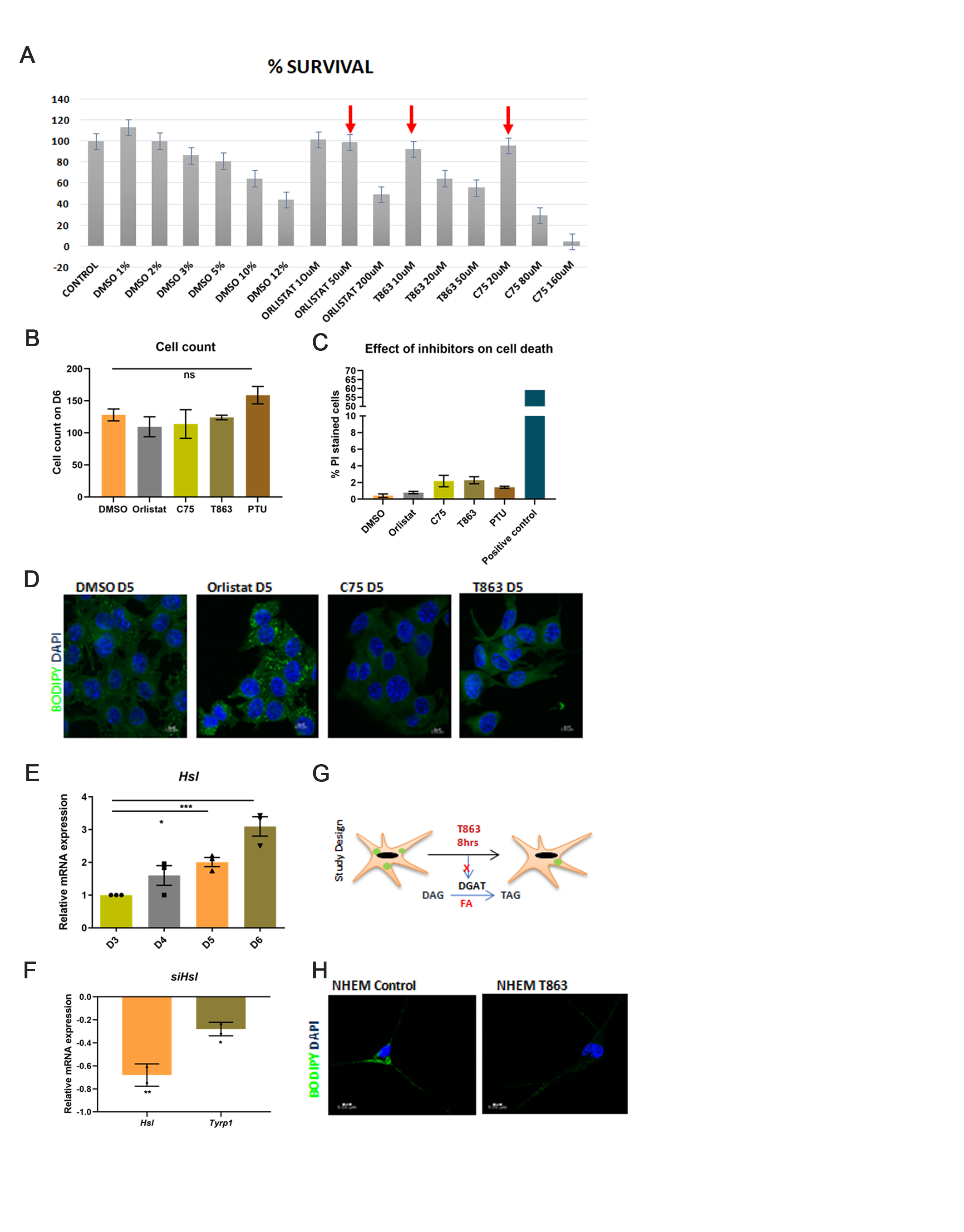

Supplement: S8 Fig — (A) Bar graph represents inhibitor dose standardization in B16 cells using MTT assay. Red arrows represent the dose chosen for further experiments. (B) Bar graph representing cell count on day 6 upon treatment with Orlistat, C75, T863, and PTU. Mean ± range is plotted for 2 biological replicates. ns is nonsignificant. One-way ANOVA is applied F(4,5) = 3.964. p-Value = 0.0922. (C) Bar graph depicts cell death induced in day 6 cells upon inhibitor treatment measured using Propidium Iodide staining. Mean ± SEM is plotted for 3 biological replicates. (D) Representative confocal microscopy images showing lipid droplet accumulation in B16 cells during pigmentation upon addition of treatments (Orlistat, C75, T863) on D3 and analysis on D5. Images were taken at 63×. Scale is 5 μm. (E) Bar graph depicting qRT-PCR of Hsl with respect to Hgprt. Mean ± SEM is plotted for 3 independent biological replicates. One-way ANOVA is applied. For Hsl, F (3,8) = 15.92, p-value = 0.001. Turkey’s test is performed for pairwise comparison. (F) Bar graph representing qRT-PCR-based quantitation of Hsl and Tyrp1 genes on D6 upon silencing of Hsl using smart pool siRNA. Mean ± SEM is plotted for 3 biological replicates. One-way ANOVA is applied, F(3,4) = 61, ***p-value = 0.0009. Turkey’s test is performed for pairwise comparison. (G) Schematic study design for analyzing TAG formation in primary human melanocyte culture by inhibiting DGAT1 using T863, and capturing lipid droplets content in these cells using BODIPY dye. (H) Representative confocal microscopy images showing lipid droplet accumulation in primary melanocytes upon T863 addition. Images were taken at 63×. Scale is 5 μm. Quantitative data are provided in S2 Data for Panels A, B, C, E, and F. PTU, 1-phenyl-2-thiourea; qRT-PCR, quantitative real-time polymerase chain reaction; siRNA, small interfering RNA; TAG, triacylglycerol. (TIF) [file pbio.3001634.s008.tif]

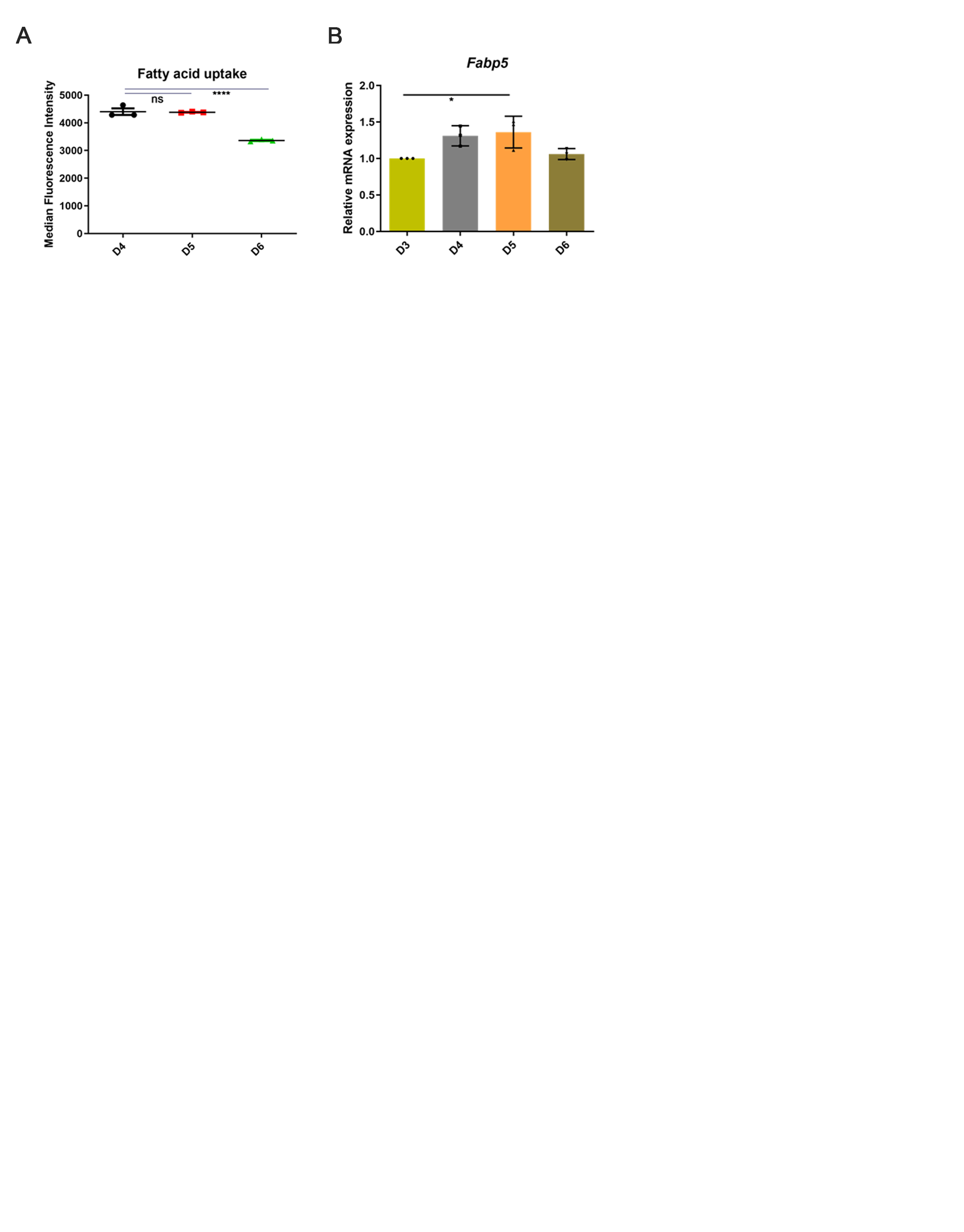

Supplement: S9 Fig — (A) Representative plot depicts median fluorescent intensity corresponding to the uptake of C-12 fluorescently labelled fatty acid from days 4 to 6. Mean ± SEM is plotted for 3 biological replicates. One-way ANOVA is applied, F(2,6) = 72.66. Turkey’s test is performed for pairwise comparison. For D4 vs. D5, p-value is nonsignificant. For D4 vs. D6, ***p-value < 0.0001. (B) Bar graph depicting qRT-PCR of Fabp5 with respect to Hgprt. Mean ± SEM is plotted for 3 independent biological replicates. One-way ANOVA is applied. Turkey’s test is performed for pairwise comparison. For Fabp5, F (3,8) = 5.374, p-value = 0.0255. ns is nonsignificant. Quantitative data are provided in S2 Data for Panels A and B. qRT-PCR, quantitative real-time polymerase chain reaction. (TIF) [file pbio.3001634.s009.tif]
