# Supplementary material for: Temporal analysis of melanogenesis identifies fatty acid metabolism as key skin pigment regulator
Source: PLoS Biol. 2022 May 18;20(5):e3001634. doi: 10.1371/journal.pbio.3001634 (PMC9116682; doi:10.1371/journal.pbio.3001634)
Supplement: S1 Raw Images — (PDF) [file pbio.3001634.s014.pdf]

Fig 1B

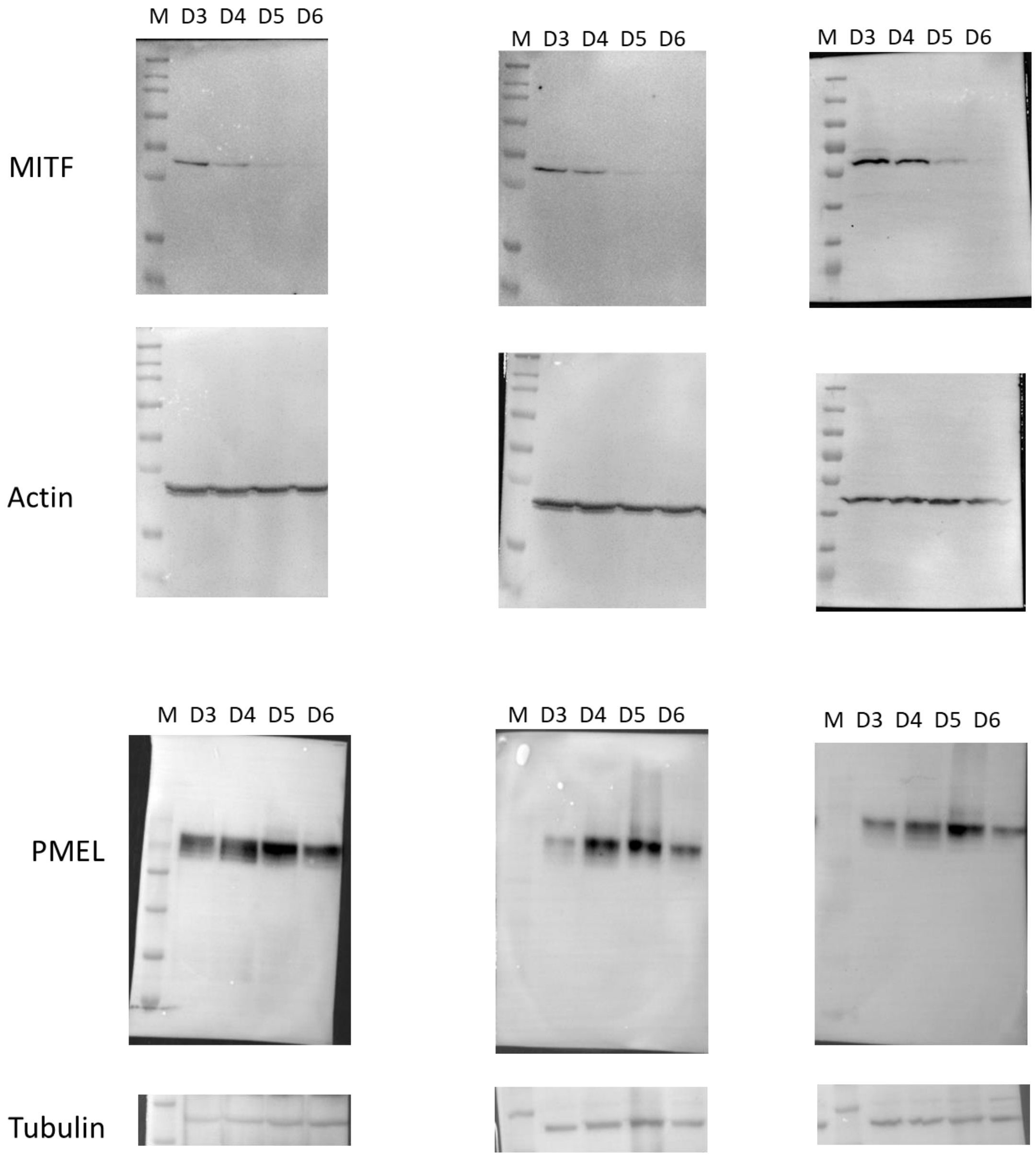
M D3 D4 D5 D6
M D3 D4 D5 D6
M D3 D4 D5 D6
MITF
Actin
M D3 D4 D5 D6
M D3 D4 D5 D6
M D3 D4 D5 D6
PMEL
Tubulin

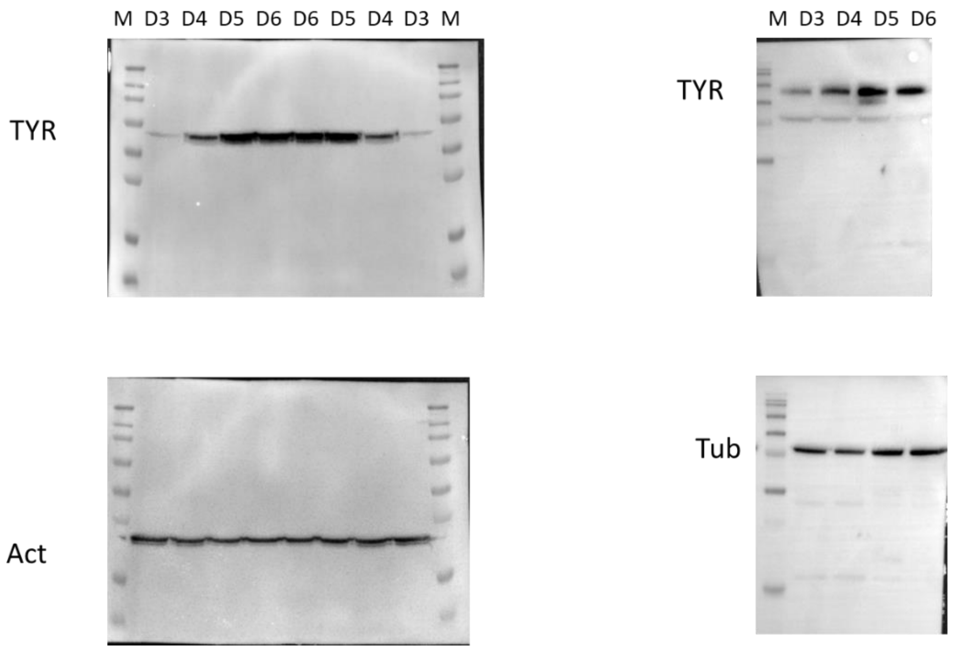
M D3 D4 D5 D6 D6 D5 D4 D3 M
TYR
Act
M D3 D4 D5 D6
TYR
Tub

Fig 5A

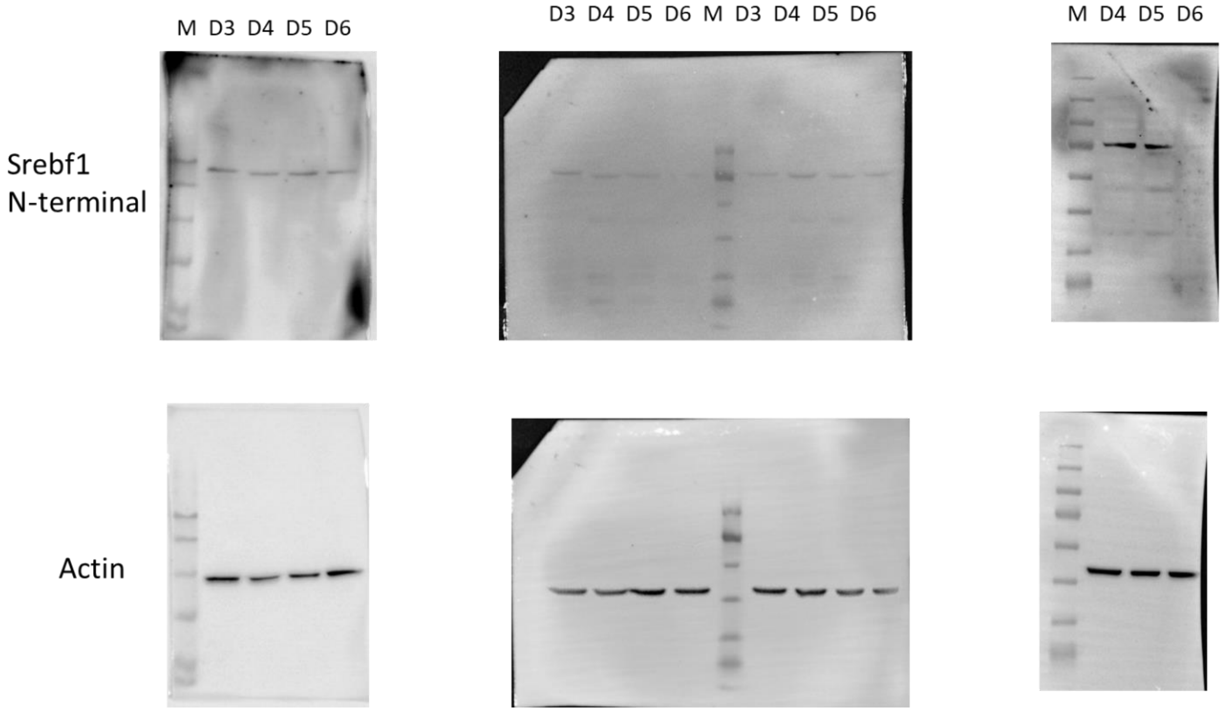
M D3 D4 D5 D6
D3 D4 D5 D6 M D3 D4 D5 D6
M D4 D5 D6
Srebf1
N-terminal
Actin

Fig 5D

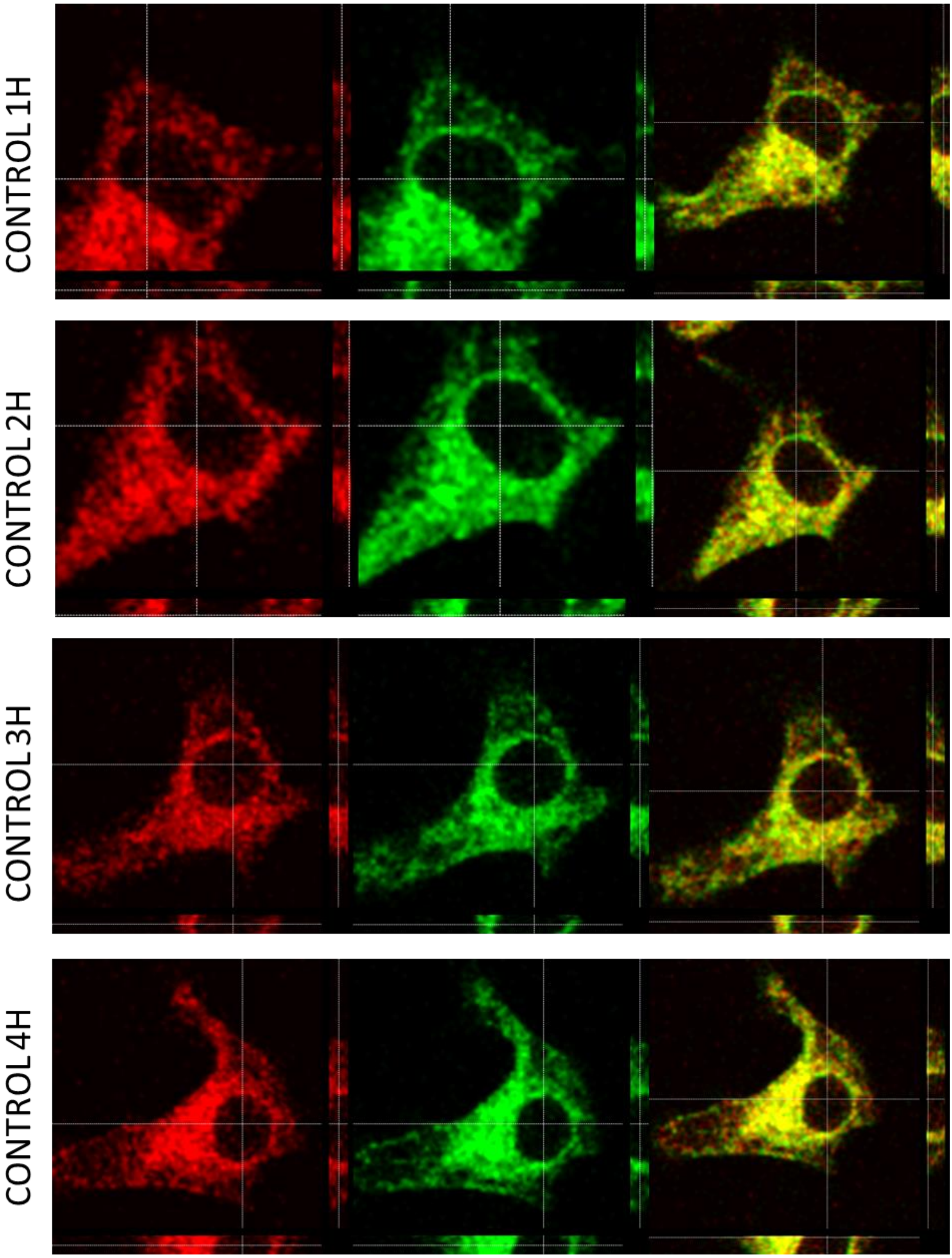

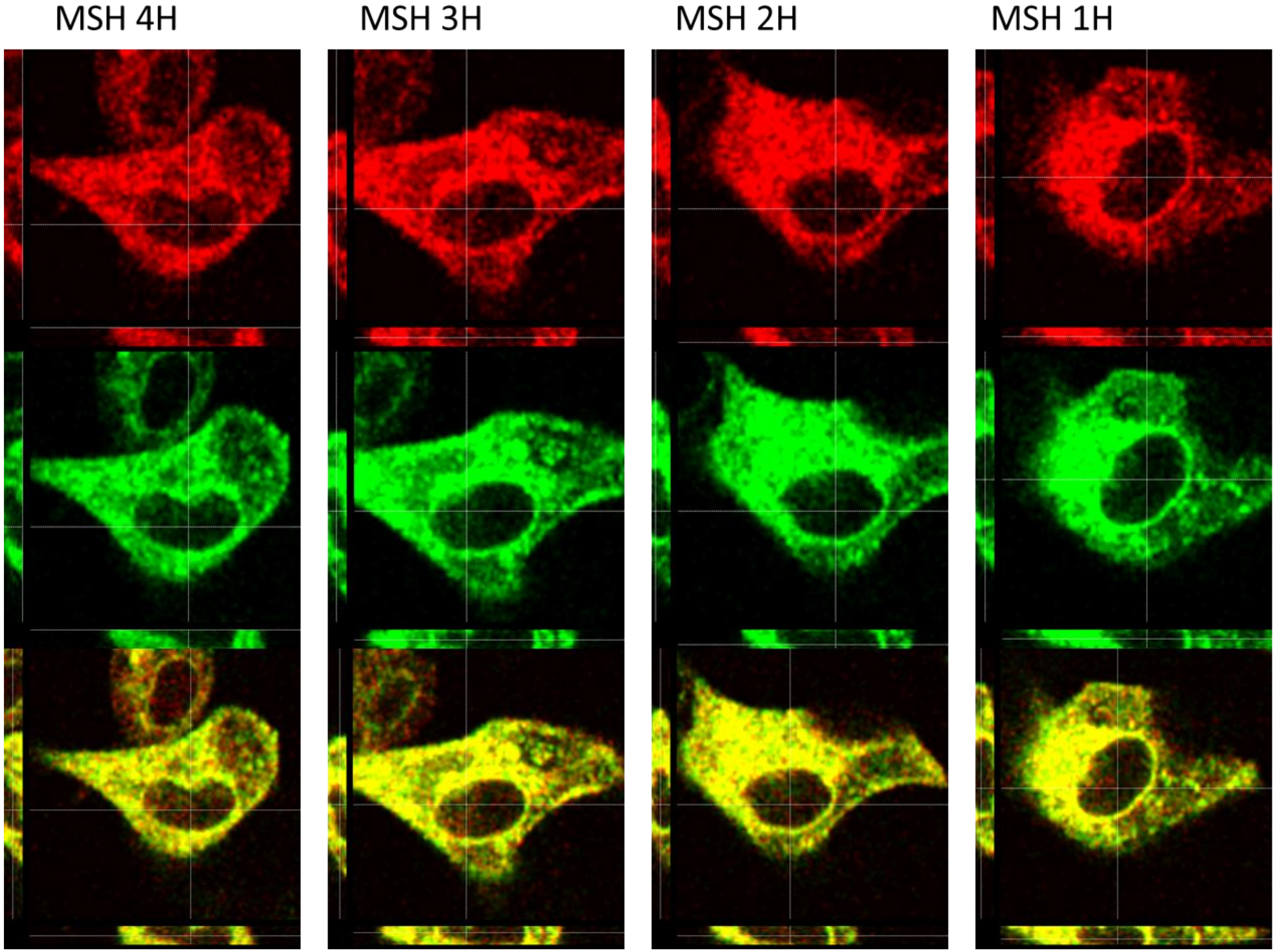
MSH 4H
MSH 3H
MSH 2H
MSH 1H

Fig 6B

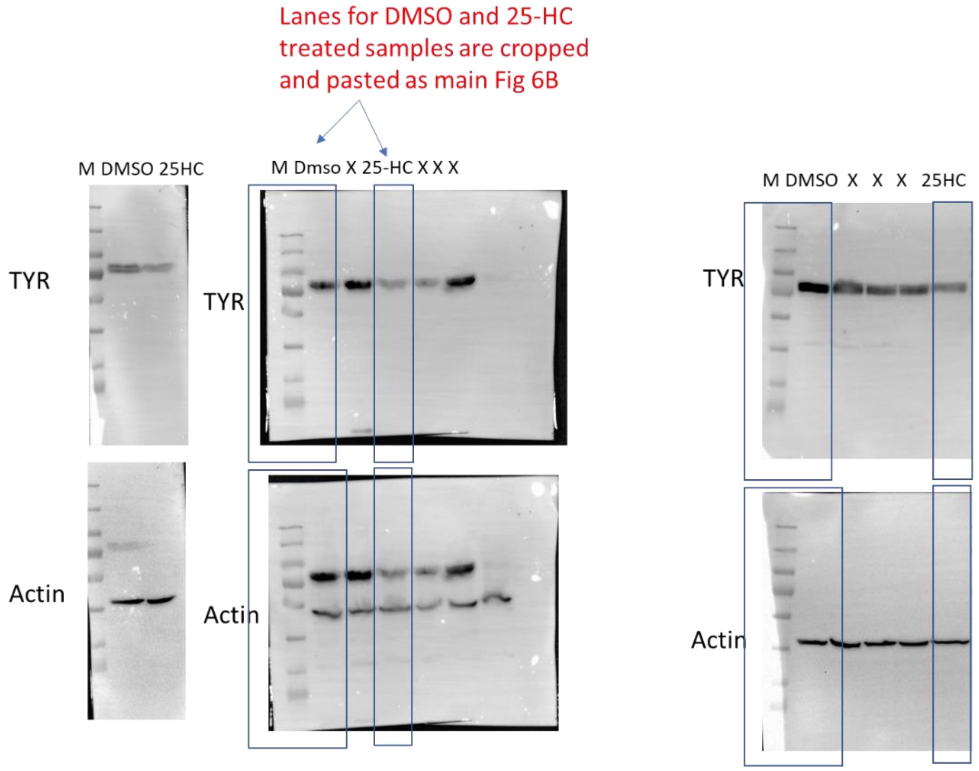
Lanes for DMSO and 25-HC treated samples are cropped and pasted as main Fig 6B
M DMSO 25HC
TYR
Actin
M Dmso X 25-HC X X X
TYR
Actin
M DMSO X X X 25HC
TYR
Actin

Fig 6G

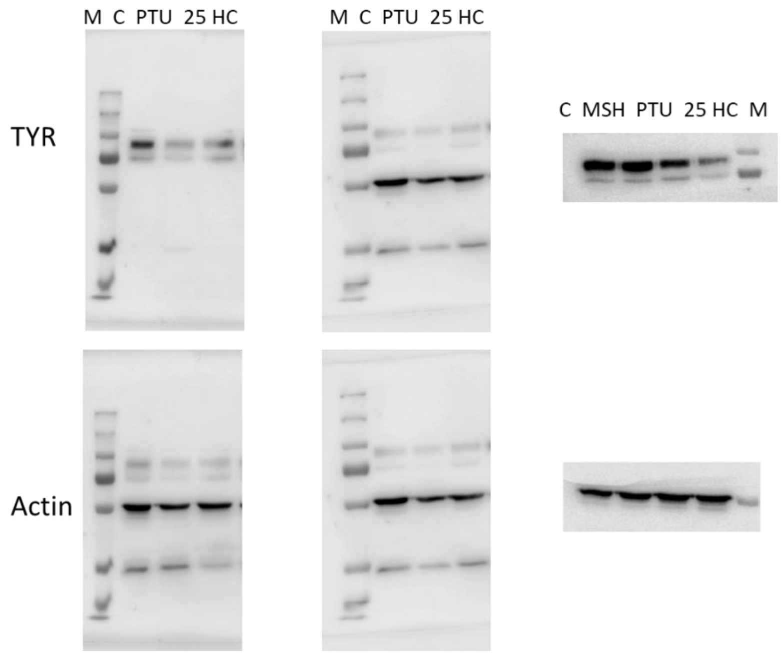
M C PTU 25 HC
TYR
Actin
M C PTU 25 HC
C MSH PTU 25 HC M

Fig 7D

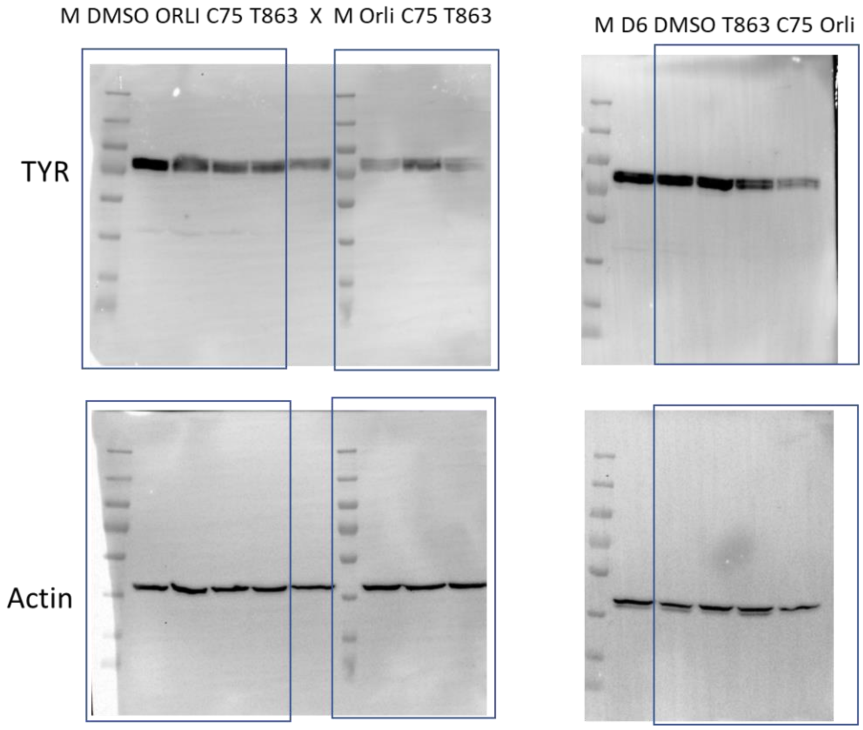


S1G Fig

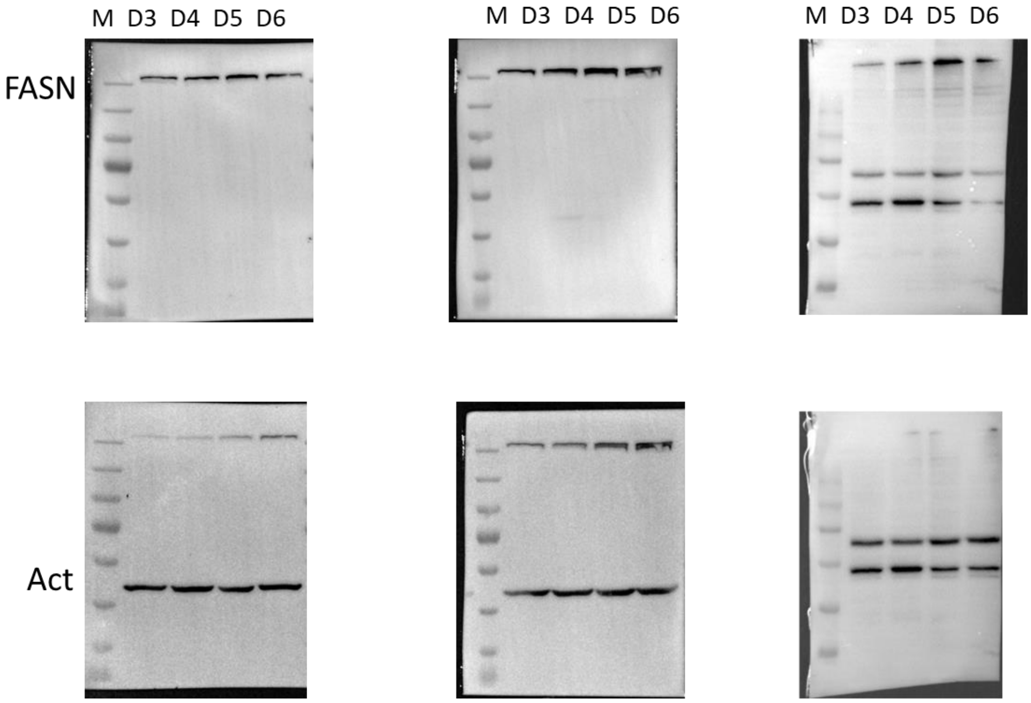

S1I Fig

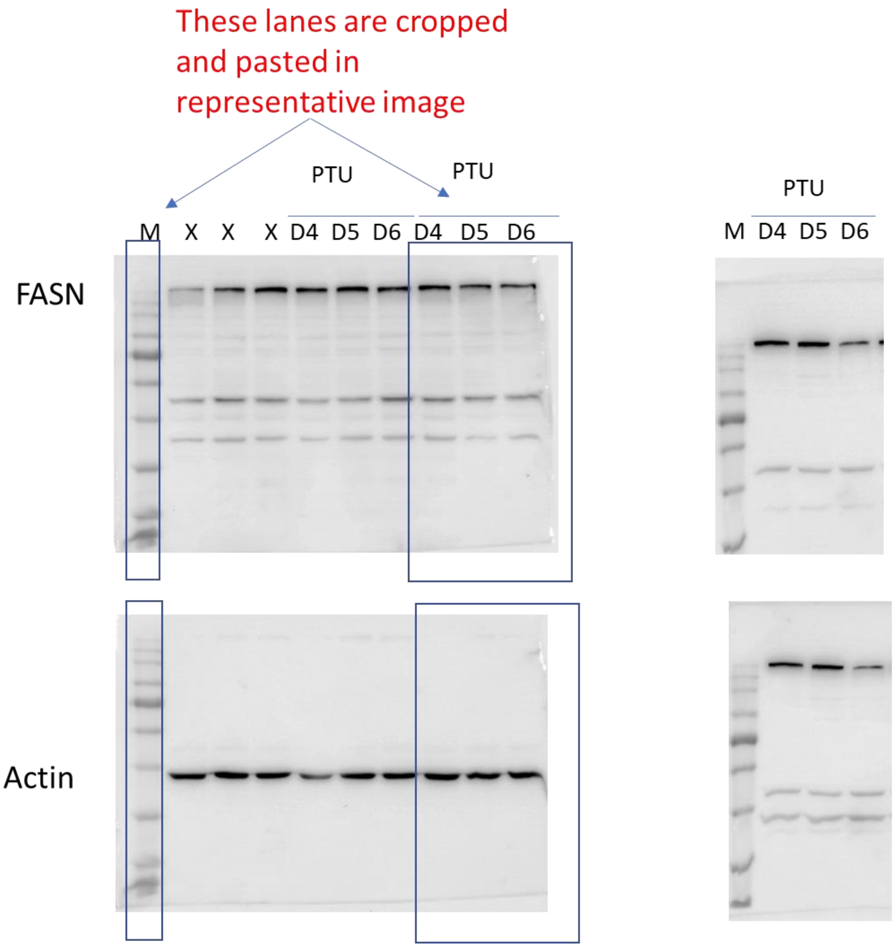


S5A Fig

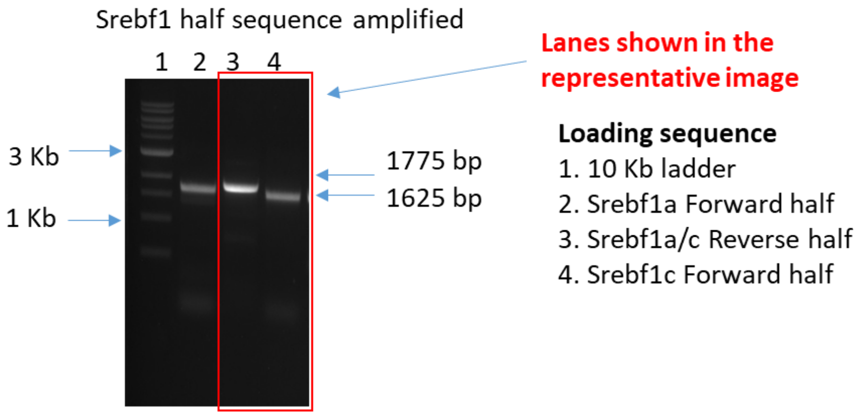

S5B Fig

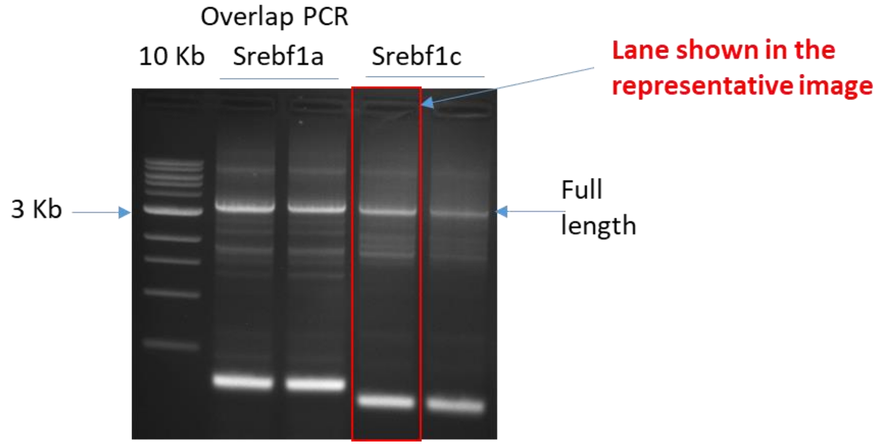


S5C Fig

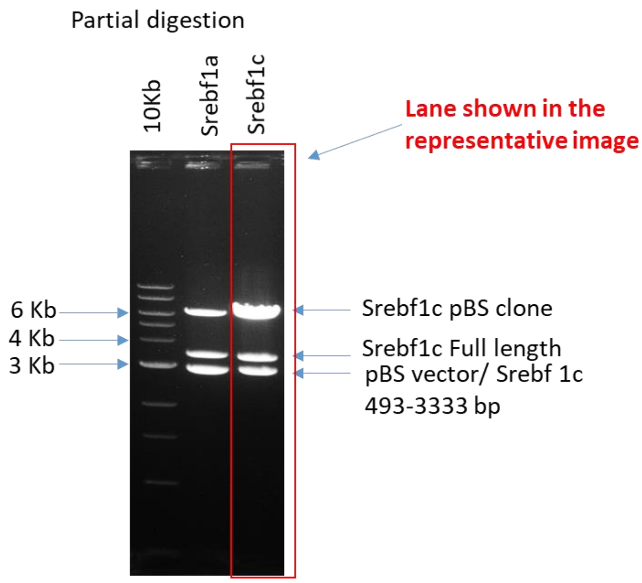


S5E Fig

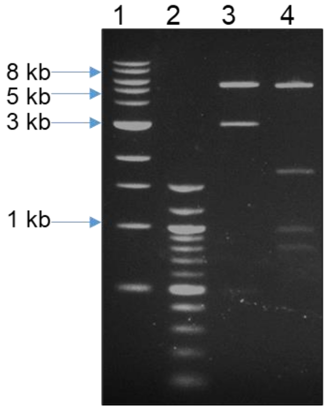


**Loading sequence**

1- 1 kb marher

2- 100 bp marker

3- pmCherry-Srebf1c-eGFP (XhoI+EcoRI)

4- pmCherry-Srebf1c-eGFP (BamHI+HindIII)

S5G Fig

| Component | Band size (kDa) |
|---|---|
| Srebf1c full-length | 126 |
| mCherry | 26 |
| eGFP | 26 |
| **Expected size** mCherry-SREBF1c-eGFP | **178** |

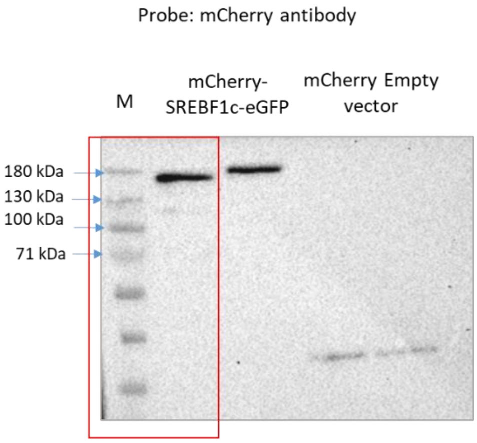


S6A Fig

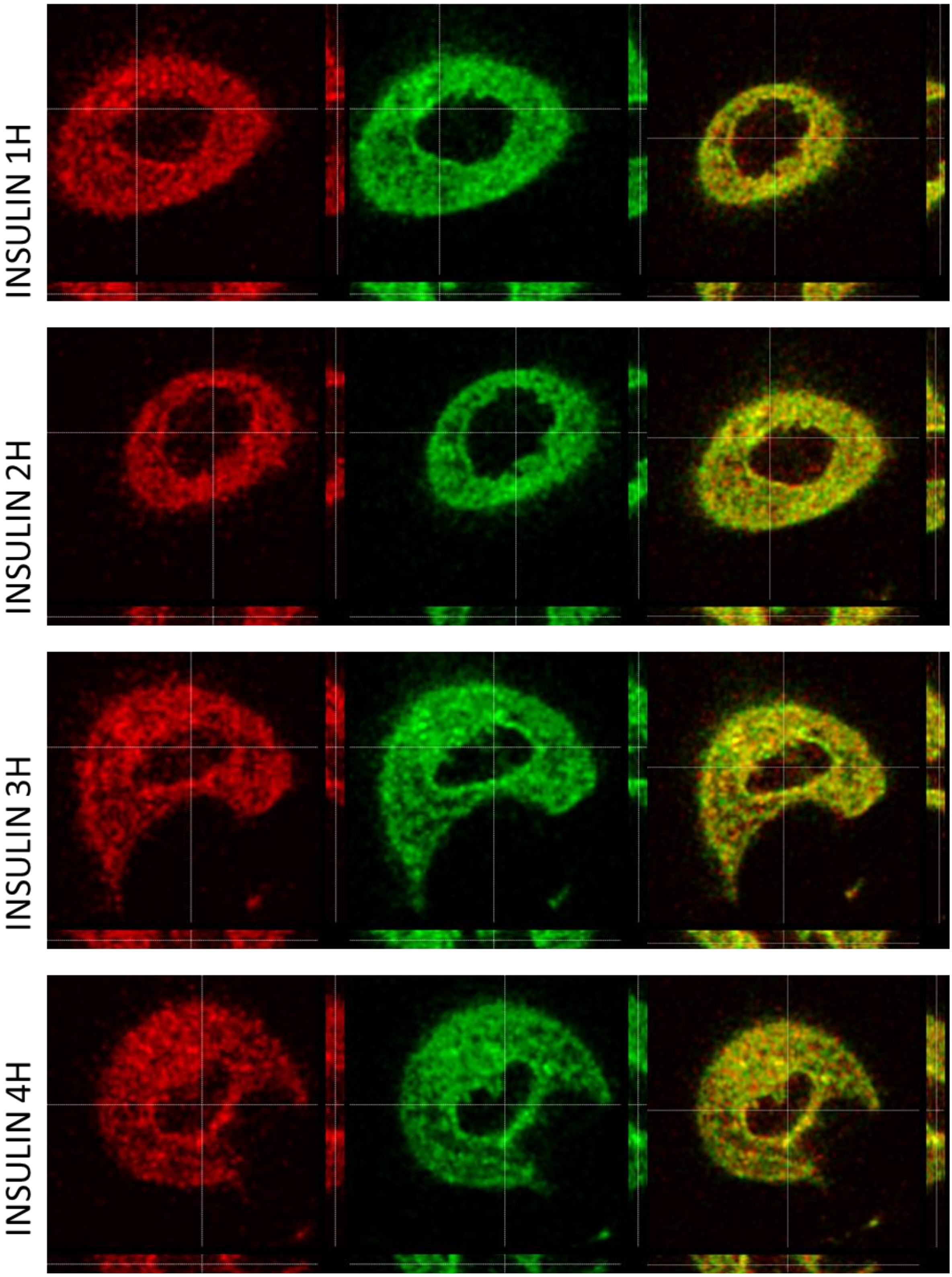
INSULIN 1H
INSULIN 2H
INSULIN 3H
INSULIN 4H
