# Supplementary material for: Temporal analysis of melanogenesis identifies fatty acid metabolism as key skin pigment regulator
Source: PLoS Biol. 2022 May 18;20(5):e3001634. doi: 10.1371/journal.pbio.3001634 (PMC9116682; doi:10.1371/journal.pbio.3001634)
Supplement: Attachment — Submitted filename: Ponit-by-point response to Reviewers comment.docx [file pbio.3001634.s015.docx]

**Point-by-point response to reviewer’s comments**

Thank you very much for the opportunity to revise our manuscript. We thank all the reviewers for their comprehensive reviews and positive feedback. We have addressed all the concerns and incorporated these changes in the manuscript. We hope that the modified manuscript is ready for acceptance and publication.

REVIEWS:

Reviewer #1, Yejing Ge: Conducting transcriptional and metabolomic profiling on a cellular pigmentation model, Sultan et al described three dynamic phases of melanogenesis, including an initial high-MITF state with rapid uptake of glucose, followed by a pigmented state accompanied by anabolic pathways and melanosome biogenesis, and a final recovery phase presumably with activation of the NRF2 detoxication pathway. During pigmented state, the authors suggest SREBF1-mediated upregulation of fatty acid synthesis results in a transient accumulation of lipid droplets and enhancement of fatty acids oxidation through mitochondrial respiration. While this heightened bioenergetic activity is important to sustain melanogenesis, it impairs mitochondria and shifts the metabolism towards glycolysis. Linking this finding to hyper-pigmentary diseases, it was further shown that inhibitors of lipid metabolism can resolve hyper-pigmentary conditions in a guinea pig UV-tanning model. Overall,
the findings are novel and interesting, the evidence presented is sufficient to support conclusion, and the manuscript is well-written. A few comments below:

***Our reply***: We thank the reviewer for the positive feedback and for finding our study interesting and novel.

Besides SREBPs, PPARs are known master regulators of lipid metabolism, particularly fatty acids synthesis and oxidation. The authors are encouraged to examine and/or discuss PPARa/b/g involvement in melanogenesis.

***Our reply***: We agree with the reviewer that PPARs are also the major class of transcriptional regulators of fatty acid synthesis and oxidation. Evaluation of their role would make the study more comprehensive.

During the course of the study, we had indeed examined the effect of PPAR-α/β/γ involvement in melanogenesis. However, since we did not observe any significant effect on pigmentation, this work was not included in the manuscript. We have now included the data for PPARs inhibitors (GW9662 for PPAR-γ, GW6471 for PPAR-α and GSK3787 for PPAR-β). In our RNA-seq analysis, we could only capture expression for PPAR-α and PPAR-γ, but not PPAR-β. Thus, we performed siRNA mediated downregulation of PPAR-α and PPAR-γ during pigmentation. Here also, we did not observe a significant effect on pigmentation genes.

Data is included in S7A-C Fig. Corresponding changes are also incorporated in the result section.

RNAseq and network computational analysis should be complemented by experimental validations, including WB and/or qPCR of differentially expressed genes and upstream transcriptional regulators.

***Our reply***: Yes, we agree that the validation would be necessary. We had carried out these validations earlier but omitted due to space constrains. We have now added the qPCR data for validation of the key genes and transcriptional regulators that emerge in our transcriptional analysis in S2 Fig. This is also included in the text as follows.

“To validate key transcriptional networks that emerged on different days in TF-TG analysis, we analysed mRNA expression of *Mitf, Srebf1, Nrf2* and their downstream target genes. Consistent with the TF-TG analysis, *Mitf* showed significant upregulation on day 3 and day 4, (S2A Fig) and *Tyr*, a rate-limiting enzyme in melanin synthesis, showed significantly higher expression on day 5 and day 6 (S2B Fig). *Srebf1* emerges as a prominent metabolic network on day 5. *Srebf1* showed maximum upregulation on day 5, followed by day 6 at mRNA level (S2C Fig). This is congruent with the observed induction up to 1.5- to 2-fold for key fatty acid synthesis genes, *Fasn*, *Acaca*, *Acacb,* and *Acly* on day 5 and day 6, as analysed by qRT-PCR (S2D Fig). Another important network that emerged on day 6 is *Nrf2*. Examination of mRNA expression of *Nrf2* and its target genes, *Gsr* and *Gst*, showed a consistent upregulation on day 6 (S2E,2F Fig). Together, TF-TG analysis delineates diverse transcriptional networks regulating metabolism, mediating an important role during the transition from depigmented to pigmented state”.

Even though the Gokhale group has well-established the low-density B16 pigmentation model in previous publications (Natarajan VT et al Proc Natl Acad Sci. 2014; Motiani RK et al EMBO J. 2018), it would be beneficial to provide more details about this assay.

***Our reply***: As suggested by the reviewers, we have described the B16 model in the beginning of the result section and added a detailed schematic in Fig 1A.

“B16 pigmentation model is a density-dependent model, wherein cells are seeded at very low density of 100 cells/cm^2^ and transition from basal depigmented to the pigmented state of cells occurs over a period of 6 days as shown in the schematic (Fig 1A). In this model, melanogenesis is triggered by the fine balance between the intrinsic needs of the cells and the constraints imposed by the extrinsic conditions. A series of coordinated processes encompassing transcriptional activation, melanosome biogenesis and melanin synthesis can be captured at the phenotypic and molecular level. The phenotypic changes in the melanin are best observed and quantitated from day 3 to day 6 (Fig 1A)”

Throughout the manuscript, quantification of WB is recommended to reflect dynamic changes of melanogenesis proteins.

***Our reply***: We have added the numerical value for mean quantitation for three blots. Mean ± SEM is plotted as a bar graph for all the replicates (Fig 1B, 1C, 5A, 5B, 6B, 6C, 6G, 6H, 7D, 7E, S1G-J).

Reviewer #2: In this paper, the authors use gene expression profiling and metabolic tracing to establish a key role for fatty acids in melanogenesis. This is a useful contribution to the field as the metabolic inputs into melanocyte differentiation are not well understood. They use a variety of pharmacologic agents to inhibit fatty acid biogenesis or metabolism to confirm their gene expression results, with limited genetic confirmation. While some of their results are correlative, it opens up downstream genetic experiments to test the hypotheses raised here.

***Our reply***: We thank the reviewer for the positive feedback.

Major points:

1.      Using both isotope tracing (Fig 3E) and Seahorse (Fig 4A-C) the authors suggest that D5 cells will increase beta-oxidation of exogenously provided fatty acids to fuel the TCA cycle. However, for much of the paper they focus on the role of fatty acid synthesis rather than uptake in providing the substrates for beta-oxidation. Could the authors explore
a.      The expression of known FA transporters in their RNA-seq data and how that compares to the expression of FA synthesis enzymes

***Our reply***: We have examined the expression of FA transporters in RNA-seq data, we find that few fatty acid transporter genes are expressed. With qRT-PCR analysis, we observed that *Fabp5* showed marginal upregulation on day 4 and day 5 and may contribute to fatty acid uptake. Data is included in S9B Fig.

Validation data for upregulation of the genes involved in the fatty acid synthesis pathway, *Acly, Fasn, Acaca, Acacb* is provided in figure S1C and S2D.

b.      Is there evidence of altered fatty acid uptake (i.e. BODIPY uptake in D5 cells) versus increased synthesis rates (i.e. labelled tracing experiments)? A FAO Seahorse assay with Palmitate could also be used to determine the contribution of extrinsic v. endogenous FAs to beta-oxidation in D5 melanocytes.

***Our reply***: Lipid droplet analysis data with BODIPY dye during pigmentation, without any oleate supplementation in media, suggests an increase in LD from endogenous fatty acid synthesis. While feeding experiments (Seahorse) and tracing experiments (^13^C Palmitate) suggest that cells can uptake fatty acid if provided in the media.

Further, we measured the fatty acid uptake ability of cells during pigmentation using C12-Bodipy labelled fluorescent fatty acids. We observed that in comparison to day 6 cells, day 4 and day 5 showed significantly higher uptake. This data is included in S9A Fig.

Together, our data suggest that both the pathways, FA uptake and *de novo* fatty acid synthesis are like to provide fatty acids for β-oxidation. Since fatty acid uptake is dependent on the availability of exogenous fatty acids, our focus was more on *de novo* fatty acid synthesis.

2.      In Figure 7A-E the authors show that inhibition of either FA synthesis or LD biogenesis will lead to decreased pigmentation. However, treatment with the inhibitor orlistat is thought to also inhibit FASN (similar to C75) but also block lipolysis leading to a failure to reduce LD content in Figure S5. However, melanin levels still decrease with orlistat treatment. Could the authors address whether lipolysis of lipid droplets can also affect pigmentation (for instance using Atglistatin) or if blocking FA synthesis is all that is required to decrease melanin content.

***Our reply***: We agree with the reviewer about the broad specificity of the Orlistat. siRNA based silencing of hormone sensitive lipase (HSL) recapitulates the depigmentation phenotype. In the follow-up work, we are indeed studying the role of lipases with more specific inhibitors. This statement is added to the discussion.

3.      In Figure 7G the representative images chosen for D0 make it appear as though even prior to treatment start pigmentation is increased in the lower half of the animal. Since the most critical comparison would be between UV treatment with and without Orlistat, and it appears that the Orlistat + UV treated group is less pigmented at baseline could the authors provide data across treatment groups such that it is clear there is a change in pigmentation regardless of the physical location of the treatment group on the animal. Alternatively the authors could remove this in vivo data since not critical to the paper.

***Our reply***: Anatomically one can observe the subtle level of pigmentation difference in the skin of Guinea Pig for different parts of their body. To make our comparison robust, we have performed experiments on the same animals in groups of three and followed them temporally. This data is now quantitated and presented in Fig 7H.

We hope the reviewer would agree with us that in the present form, data is reasonably robust and shows quantitative differences.

4.      Figure 4 shows that at D6 the cells have a significant increase in damaged mitochondria. Could the authors provide additional data/explanation of why this might be the case, is this typical of highly differentiated cells? Could mitochondrial fragmentation be due to a decrease in cellular fitness, i.e. increased cell death/senescence?

***Our reply***: To address that mitochondrial fragmentation observed on day 6 is due to pigmentation, we have compared mitochondrial fragmentation in depigmented day 6 cells using phenylthiourea (a known pigmentation inhibitor). Data is included in S4A,4B Fig. Further, pigmented and depigmented cells on day 6 shows comparable cell death in range of 1-2 percent (DMSO vs PTU data in S8C), suggesting the mitochondrial changes are attributed by pigmentation. We have included the following statement in the text.

“To determine that the observed mitochondrial changes are attributed to pigmentation and not senescence, we analyze the mitochondrial morphology in D6 pigmented cells and PTU- treated D6 depigmented cells using Mitotracker RED dye (S4A Fig). Analysis of individual mitochondria using Image J macro tool for Mitochondrial Network Analysis (MiNA) showed a significantly high round and punctate mitochondria in pigmented D6 cells (S4B Fig).”

5.      Figure 6 relies heavily on the use of 25-HC to block srebf1, however, 25-HC is known to have other effects outside of srebp inhibition. While specific pharmacologic inhibition of srebf1 may be difficult could the authors comment on the specificity of 25-HC for srebf1 in the text. Futhermore, are there other ways to more specifically target this pathway, for instance PPAR inhibitors?

***Our reply***: We have cited the literature about the specificity of 25-HC in our manuscript as “25-hydroxycholesterol shows broad specificity for both *Srebf1* and *Srebf2* (37,38).”

To substantiate the specific role of *Srebf1*, we show that siRNA mediated downregulation of *Srebf1* gene, results in decreased pigmentation and expression of genes involved in the pigmentation pathway in B16 as well as primary melanocytes in figure 6D, 6I,6J.

We had indeed previously examined the effect of PPAR-α/β/γ involvement in melanogenesis. However, since we did not observe any significant effect on pigmentation, this work was not included in the manuscript. We have now included the data for PPARs inhibitors (GW9662 for PPAR-γ, GW6471 for PPAR-α and GSK3787 for PPAR-β) in S7A Fig. This is further substantiated by our RNA-seq analysis, where expression for PPAR-α and PPAR-γ can only be detected, but not PPAR-β. We performed siRNA mediated downregulation of PPAR-α and PPAR-γ during pigmentation. Here also, we could not observe a significant effect on pigmentation genes. Data is now included in S7B,7C Fig.

Minor points:

1.      Could the authors provide additional explanation of the pigmentation model that they chose. For instance, a schematic showing the assay setup and temporal resolution of pigmentation changes.

As suggested by the reviewers, we have described the B16 model in the beginning of the result section and added a detailed schematic in Fig 1A.

“B16 pigmentation model is a density-dependent model, wherein cells are seeded at very low density of 100 cells/cm^2^ and transition from basal depigmented to the pigmented state of cells occurs over a period of 6 days as shown in the schematic (Fig 1A). In this model, melanogenesis is triggered by the fine balance between the intrinsic needs of the cells and the constraints imposed by the extrinsic conditions. A series of coordinated processes encompassing transcriptional activation, melanosome biogenesis and melanin synthesis can be captured at the phenotypic and molecular level. The phenotypic changes in the melanin are best observed and quantitated from day 3 to day 6 (Fig 1A)”

2.      In Figure 1D the authors do not define how they performed differential gene expression analysis. Could they state which comparisons they used to generate the fold changes represented in the heat map.

***Our reply***: We have included the following statement in the main manuscript and figure legend to clarify how differential gene expression analysis was performed.

“Time course analysis was performed using the LRT test (Likelihood-ratio test) and genes with adjusted p-value < 0.001 were taken as significant differentially expressed genes from day 3 to day 6 using DESeq2. Heatmap is plotted for these 1493 differentially expressed genes obtained in time-course analysis (Fig 1D).”

3.      In Figure 1G-H statistical analysis of the significance of LD size/number is needed. This would also support whether the claim on lines 165-166 regarding rapid depletion of LDs at D6 is true.

***Our reply***: We have now provided statistical significance for this data set in Fig 1G and 1H and included the following statement in the text.

“Quantitative analysis of the volume of lipid droplets per cell and volume of individual lipid droplets revealed the formation of these lipid aggregates significantly increases on D5 (Fig 1G, 1H). Further, a significant decrease in volume of lipid droplets per cell was observed on D6.”

4.      Could the authors provide clearer data on why they chose to focus on the presence of lipid droplets as a facet of fatty acid metabolism; specifically which genes known to be involved in LD biogenesis are upregulated in D5/D6 cells?

***Our reply***: B16 cells are not known to store lipid droplets and thus transient accumulation of lipid droplets during pigmentation is reflective of lipid metabolism changes during pigmentation. We have analysed the RNA-seq data set regarding the expression of DGATs, AGPATs and GPATs genes involved in LD biogenesis during pigmentation and provided in S1D Fig and the following statement is included in the main text as follows.

“We analysed the expression of fatty acid synthesis genes and TAG synthesis genes from RNA sequencing data (S1C,1D Fig). Analysis showed a trend of increased expression of several of the genes in both fatty acid and TAG synthesis pathway with pigmentation. This may result in increased lipid droplets formation during pigmentation.”

5.      Inclusion of Figure 1I-J is confusing in this location. The data might be better suited to be presented when the authors examine the effect of the DGAT inhibitor as well as FA synthesis inhibitors on melanin content in Figure 7.

***Our reply***: As per the suggestion, we will remove this data from figure 1 and place it along with the inhibitor data in S8E,8F Fig.
