# Supplementary material for: Temporal analysis of melanogenesis identifies fatty acid metabolism as key skin pigment regulator
Source: PLoS Biol. 2022 May 18;20(5):e3001634. doi: 10.1371/journal.pbio.3001634 (PMC9116682; doi:10.1371/journal.pbio.3001634)
Supplement: Attachment — Submitted filename: Ponit-by-point response to Reviewers comment.docx [file pbio.3001634.s016.docx]

**Point-by-point response to reviewer’s comments**

We thank the reviewers and editors for their positive feedback and the opportunity to revise our manuscript. We have addressed all the concerns and incorporated these changes in the manuscript. We hope that the modified manuscript is ready for acceptance and publication.

Rev. 1:

The revised manuscript has fully addressed my concerns and is acceptable for publication.

**Our Reply**: We thank you for finding our study acceptable for publication.

Rev. 2:

Overall, the authors have addressed most of our initial comments. A few small remaining points:

**Our Reply:** We thank you for your positive reply and your further concerns are addressed in the revised version.

1) The authors reference the HSL data but do not include the data in the paper although it is in the methods. This data should be included and discussed.

**Our Reply:** We agree with the reviewer about the broad specificity of the Orlistat. siRNA-based silencing of hormone-sensitive lipase (HSL) recapitulates the depigmentation phenotype. Data is now included in S8E, F Fig and corresponding changes are made in the text.

“As inhibition of lipases with Orlistat showed a decrease in pigmentation, we further analyze the expression of lipase during pigmentation. We observed that the expression of one of the lipases, hormone-sensitive lipase (HSL), increases with pigmentation (S8E Fig). Moreover, a 60-70% knockdown of *Hsl* with siRNA showed a significant decrease in *Tyrp1* expression by 25-30% (S8F Fig). Altogether, inhibitor data suggests that both fatty acid synthesis and lipolysis arm are important during pigmentation for increasing availability of free fatty acids for further utilization.”

2) The pigmentation effect of srebf1 knockdown is very modest and not really quantified. It seems like the effect of this gene alone is much smaller than the inhibitor. This should be discussed in the discussion - is it compensation? Ineffective knockdown?

**Our Reply:** Following text is added to the discussion.

“While lipid metabolism is governed by PPARs and SREBFs, only *Srebf1* knockdown displayed a reproducible modest depigmenting phenotype in melanocytes, in both B16 and primary melanocytes. Although the reasons for this phenotype are unclear, it may be noted that *Srebf1* protein is cleaved and translocated to the nucleus for transcriptional activation, and the transient decrease in its mRNA expression may not be sufficient to completely reverse the pigmentation phenotype. Indeed, the inhibitor 25-HC that targets the activation of SREBF1 shows a better depigmenting effect during the course of treatment. Another possibility is the compensation effect by two Srebf1 isoforms*,* *Srebf1a* and *Srebf1c*.”
